# A Framework of Algorithms: Computing the Bias and Prestige of Nodes in Trust Networks

**DOI:** 10.1371/journal.pone.0050843

**Published:** 2012-12-11

**Authors:** Rong-Hua Li, Jeffrey Xu Yu, Xin Huang, Hong Cheng

**Affiliations:** Department of Systems Engineering & Engineering Management, The Chinese University of Hong Kong, Sha Tin, N.T., Hong Kong; Universitat Rovira i Virgili, Spain

## Abstract

A trust network is a social network in which edges represent the trust relationship between two nodes in the network. In a trust network, a fundamental question is how to assess and compute the bias and prestige of the nodes, where the bias of a node measures the trustworthiness of a node and the prestige of a node measures the importance of the node. The larger bias of a node implies the lower trustworthiness of the node, and the larger prestige of a node implies the higher importance of the node. In this paper, we define a vector-valued contractive function to characterize the bias vector which results in a rich family of bias measurements, and we propose a framework of algorithms for computing the bias and prestige of nodes in trust networks. Based on our framework, we develop four algorithms that can calculate the bias and prestige of nodes effectively and robustly. The time and space complexities of all our algorithms are linear with respect to the size of the graph, thus our algorithms are scalable to handle large datasets. We evaluate our algorithms using five real datasets. The experimental results demonstrate the effectiveness, robustness, and scalability of our algorithms.

## Introduction

In recent years, trust social networks such as Advogato (www.advogato.org), Kaitiaki (www.kaitiaki.org.nz), Epinions (www.epinions.com), and Slashdot (www.slashdot.org) rapidly attract more and more attention in both research and industry communities. In trust networks, users express their trust to other users by giving a trust score to another, and users are evaluated by others based on their trust scores. There are two types of trust networks, namely, unsigned and signed. In the unsigned trust networks, such as Advogato and Kaitiaki, users can only express their trust to other users by giving a non-negative trust score to others. In the signed trust networks, such as Epinions and Slashdot, users are able to express their trust or distrust to others by giving a positive or negative trust score to others. There are many applications in the trust networks, such as finding the trusted nodes in a network [1], predicting the trust score of the nodes [2], and the trust based recommendation systems [3], [4]. It is worth mentioning that the trust network studied here is unlike the problem of trust management [5]–[8] which is extensively studied in the literature. For example, Richardson et al. [9] proposed an eigenvector based algorithm for trust management in semantic web. Independent to Richardson's work, Kamvar et al. [10] presented a similar eigenvector based algorithm, namely Eigentrust, for trust management in P2P networks. Guha et al. [11] studied the problem of propagation of trust and distrust in the networks. Subsequently, Theodorakopoulos et al. [12] proposed a trust evaluation metric from an algebra viewpoint. They used semiring to express a trust model and then modeled the trust evaluation problem as a path problem on a directed graph. Andersen et al. [13] proposed an axiomatic approach for trust measurements. Recently, Richters and Peixoto [14] studied the trust propagation problem on social networks based on a metric of transitivity. Most of these studies are based on an *implicit* trust network, where the trust scores are estimated by some ad hoc methods. However, in trust social networks, the trust scores are explicitly given by the users.

In a signed/unsigned trust network, the final trustworthiness of a user is determined by how users trust each other in a global context, and is measured by *bias*. The bias of a user reflects the extent up to which his/her opinions differ from others. If a user has a zero bias, then his/her opinions are 100% unbiased and can be 100% taken. Consequently, the user has high trustworthiness. On the other hand, if a user has a large bias, then his/her opinions cannot be 100% taken because his/her opinions are often different from others. Therefore, the user has low trustworthiness. Another important measure, the *prestige* of a user, reflects how he/she is trusted by others (the importance). In this work, we study how to assess and compute the bias and prestige of the users. The challenges are: (1) how to define a reasonable bias measurement that can capture the bias of the users' opinions, (2) how to handle the negative trust scores in signed trust networks, and (3) how to design a robust algorithm that can prevent attack from some adversarial users.

As pointed out in [1], the classic eigenvector based methods [15], such as eigenvector centrality [16], HITS [17], and PageRank [18]–[20], cannot be directly used to solve this problem. The reason is because these methods cannot handle the negative edges, which exist in signed networks [21]. More recently, many PageRank and HITS variants, such as the PageTrust [22], the signed spectral ranking [23], reputation-based ranking [24] and the PolarityRank [25], [26] algorithms, have been extended to the case of signed networks. All of these variants, however, cannot compute the bias of the nodes. To the best of our knowledge, the algorithm proposed by Mishra and Bhattacharya [1] is the only algorithm that addresses to compute both prestige and bias of nodes in trust social network. We refer to this algorithm as the MB algorithm (or simply MB). MB is tailored for the signed trust networks, and can also be used for the unsigned trust networks. However, MB has major drawbacks. The trustworthiness of a user cannot be trusted due to the fact that MB treats bias of a user by relative differences between itself and others. For instance, if a user gives all his/her friends a much higher trust score than the average of others, and gives all his/her foes a much lower trust score than the average of others, such differences cancel out, which leads to a zero bias for the user. This cancelation happens in either a signed or a unsigned trust network. Therefore, MB can be attacked by the adversarial users. We will analyze it in the next section in detail.

In this paper, we propose new bias measurements to capture the bias of the users' opinions. First, we define a vector-valued contractive function as a framework to represent the bias vector, which implies a rich family of bias measurements and thereby results in a rich family of algorithms. On the basis of our framework, we develop four new bias measurements using absolute differences instead of relative differences to deal with bias, in order to avoid such a cancelation problem in MB. Based on the bias of the nodes, the trustworthiness score of a node is inversely proportional to the bias score of the node, and the prestige of a node is the average trustworthiness-weighted trust scores. In other words, if a node is with a large bias score, then the trust scores given by this node will be assigned to small weights. Our algorithms iteratively refine the bias and prestige scores of the nodes. The final bias and prestige vector is obtained when the algorithm converges. The major advantages of our algorithms are as follows. First, the bias measurement of our new algorithms are more reasonable, more effective, and more robust than the MB algorithm. Second, similar to MB, our algorithms can also work on signed trust networks. Third, the time and space complexity of all our algorithms are linear with respect to (w.r.t.) the size of the networks, thereby they are scalable to handle large trust networks.

## Methods

We model a trust network as a directed weighted graph 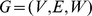 with 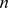 nodes and 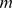 edges, where 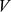 represents the node set, 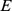 denotes the edge set, and 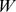 denotes the weights. In graph 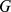, a weight 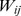 signifies a trust score from node 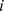 to node 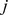. All trust scores are normalized in the range of 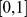. For simplicity, in the following discussions, we focus on an unsigned trust network assuming that all edge-weights are non-negative. Our approaches can be readily generalized to signed trust networks, and we will discuss it at the end of this section.

**Figure 1:**
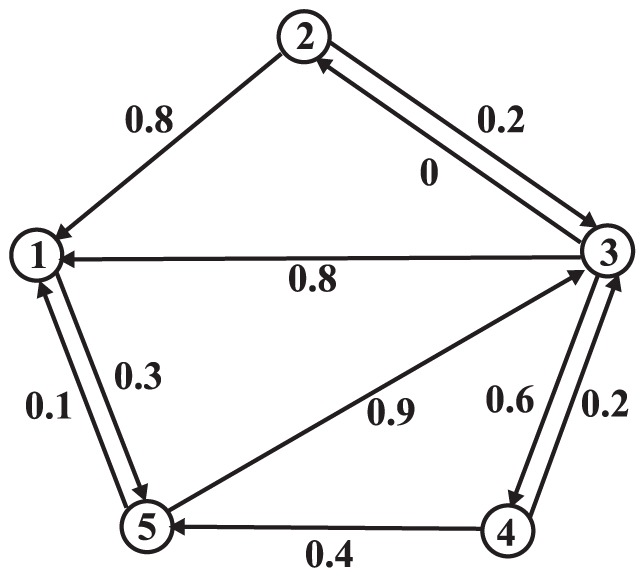
A trust network. A circle denotes a node, an arrow represents a trust relationship between two nodes, and the associated weight denotes the trust score.

An example is shown in Figure 1. In Figure 1, node 5 gives a trust score 0.1 to node 1 (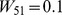), whereas two nodes, 2 and 3, give a high trust score 0.8 to node 1 (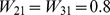). And node 5 gives a trust score 0.9 to node 3 (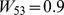), while two nodes, 2 and 4, give a low trust score to node 3 instead (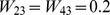). This observation shows that node 5's opinions often differ from those of others, thus indicates that node 5 is a biased node. On the other hand, there are two nodes (2 and 3) giving a high trust score 0.8 to node 1 (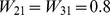), which suggests that node 1 would be a prestigious node. Additionally, in this example, node 3 gives 0 to node 2 (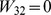), which implies that node 3 does not trust node 2 at all.

Given a trust network 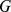, the problem we study is how to compute the bias and prestige of the nodes based on the trust scores. As discussed, the eigenvector based methods are not applicable, and the only existing solution is MB [1]. In the following, we briefly review MB and discuss its major drawbacks.

In MB, each node has two scores: the bias and prestige score. The bias and prestige scores of node 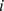 are denoted by 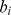 and 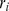, respectively. Formally, the bias of node 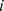 is defined by
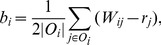
(1)where 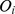 denotes the set of all outgoing neighbors of node 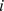. The idea behind is that a node will be assigned to a high bias score if it often behaves differently from others. The prestige score of node 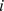 (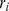) is given by
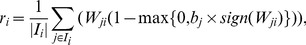
(2)where 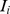 denotes the set of all incoming neighbors of node 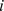, and 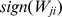 denotes the sign of an edge from node 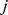 to node 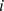, which can be positive (trust) or negative (distrust).

The MB algorithm works in an iterative fashion, and the corresponding iterative system is
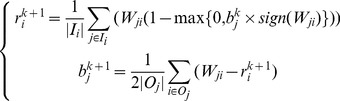
(3)


There are two major drawbacks in MB. First, in Eq. (1), the differences (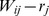) for different outgoing neighbors 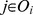 can be canceled out, thus will result in unreasonable bias measures. Reconsider the example (Figure 1), node 5 gives 0.1 to node 1, while both node 2 and node 3 give 0.8 to node 1. With these three edges (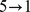, 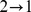, and 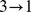), the trust score given by node 5 is significantly lower than those of others with a difference 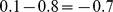. However, consider the other three edges 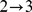, 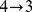, and 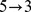, we can find that the trust score given by node 5 is significantly larger than those of the other two nodes (nodes 2 and 4) with a difference 0.7. The positive and negative differences can be canceled out by Eq. (1), and this will cause node 5 to be trusted with a lower bias score. However, intuitively, node 5's opinions often differ from those of others, thereby it should be assigned to a large bias score. Table 1 shows the bias scores by MB after each iteration. We can clearly see that node 5 gets the minimal bias scores (0.014), which contradicts to the intuition.

**Table 1:** Bias scores by the MB algorithm.

Iteration	node 1	node 2	node 3	node 4	node 5
1	0.350	0.042	0.121	0.250	0.042
2	0.350	0.015	0.129	0.232	0.015
3	0.350	0.014	0.129	0.231	0.014
4	0.350	0.014	0.129	0.231	0.014

The table shows the bias scores by the MB algorithm in the trust network given in Figure 1. The MB algorithm converges in 4 iterations. Note that node 5 achieves the lowest bias score.

Second, as also pointed in [1], MB is easy to be attacked by the adversarial nodes. For example, some nodes can maintain their bias scores closely to 0 by giving high trust scores to the nodes with low prestige scores and giving the low trust scores to the nodes with high prestige scores (as node 5 in Figure 1). In [1], Mishra and Bhattacharya present a statistical method for detecting such adversarial nodes. But the statistical method is independent to MB, thus it cannot reduce the influence of the adversarial nodes in MB. In addition, the proof for the convergence of the MB presented in [1] is not rigorous. In the present paper, we rigorously prove the convergence of our framework using the Cauthy convergence theorem [27].

### Our New Approach

Here we propose a framework of algorithms for computing the bias and prestige of the nodes in trust networks. In our framework, every node 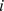 has two scores: the bias score (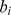) and the prestige score (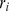). We use two vectors 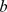 and 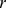 to denote the bias vector and prestige vector, respectively. Specifically, we define the bias of node 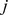 by
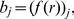
(4)where 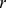 is the prestige vector of the nodes, 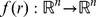 is a *vector-valued contractive function*, which is defined in Definition 0, and 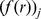 denotes the 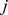-th element of vector 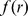. We restrict 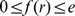, where 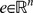 and 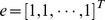.

#### Definition 1

For any 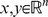, the function 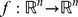 is a vector-valued contractive function if the following condition holds.
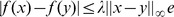
(5)where 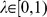, 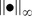 denotes the infinity norm.

Since 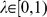, the vector-valued function 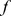 exhibits contractive property w.r.t. the infinity norm of the vector, we refer to it as the vector-valued contractive function. It is worth noting that the vector-valued contractive function we define is a generalization of the contraction mapping in the fixed point theory [28]. In [28], the contraction mapping is defined on a 1-dimensional variable and the domain of the contraction mapping is also a 1-dimensional value. Our vector-valued contractive function is defined on an 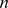-dimensional vector and its domain is also an 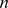-dimensional vector. The contraction mapping is very useful for iterative function systems [28]. Our vector-valued contractive function sheds light on studying the iterative vector-valued function systems in trust networks.

As can be seen in Eq. (4), the bias vector 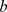 is obtained by a vector-valued contractive function defined on the prestige vector 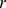. The advantage of the definition of bias is that it makes our framework general, which will result in a rich family of bias measurements. Later, we will give four different bias measurements and each of these measurements is shown to be a vector-valued contractive function.

With the bias of the nodes, the trustworthiness of node 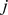 is given by 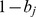, which is inversely proportional to the bias score of node 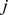. We compute the prestige score of node 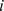 by averaging the trustworthiness-weighted trust scores given by the incoming neighbors of node 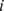. In particular, the prestige score 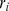 for a node 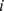 is given by
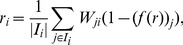
(6)where 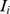 is the set of all incoming neighbors of node 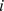. Our framwork iteratively refines the prestige vector and the bias vector using the following iterative system:
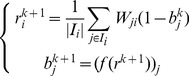
(7)where 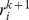 denotes the prestige of node 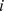 in the 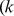
+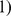-th iteration and 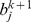 denotes the bias of node 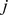 in the 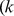
+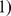-th iteration. Initially, we set 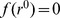, which implies 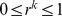. The iterative system defined in Eq. (7) converges into a unique fixed prestige and bias vector in an exponential rate of convergence. The detailed convergence analysis of the proposed approach can be found in File S1.

### Instances of 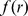


Here we first show that MB is a special instance of our framework on unsigned trust networks. Then, based on our framework, we present four new algorithms that can circumvent the existing problems of MB.

To show that MB on the unsigned trust network is a special instance of our framework, we show that 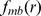 is a vector-valued contractive function. The 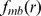 is defined by
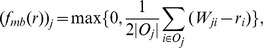
for 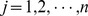. In particular, we have the following theorem. All the proofs can be found in File S1.

#### Theorem 1

*For any* 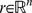, *and* 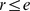, 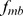 *is a vector-valued contractive function with the decay constant* 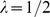 *and* 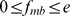.

As analysis in the previous section, MB yields unreasonable bias measurement and it is easy to be attacked by the adversarial nodes. In the following, we propose four new algorithms that can tackle the existing problems in MB. Specifically, we give two classes of vector-valued contractive functions: the 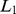 distance based vector-valued contractive functions and the 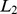 distance based vector-valued contractive functions. All functions can be served as 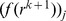 in Eq. (7). That is to say, all of these functions can be used to measure the bias of the nodes.

#### 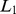 distance based contractive functions

We present two vector-valued contractive functions based on the 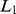 distance measure: 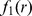 and 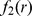. Specifically,
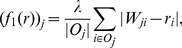
(8)for all 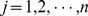. In the following theorem, we show that 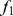 is a vector-valued contractive function.

#### Theorem 2

*For any* 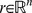, *and* 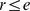, 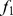 *is a vector-valued contractive function with* 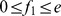.

Based on 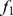, the bias of node 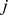 is determined by the arithmetic average of the differences between the trust scores given by node 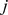 and the corresponding prestige scores of the outgoing neighbors of node 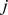. The rationale is that the nodes whose trust scores often differ from those of other nodes will be assigned to high bias scores. In 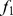, the difference is measured by the 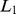 distance, thus we refer to this algorithm as the 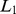 average trustworthiness-weighted algorithm (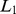-AVG). The corresponding iterative system is given by
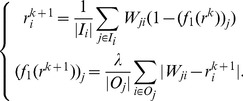
(9)


It is important to note that, unlike MB, 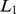-AVG uses the 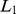 distance to measure the differences, thus the differences between the trust score and the corresponding prestige score cannot be canceled out. It therefore can readily prevent attacks from the adversarial nodes that give the nodes with high prestige low trust scores and give the nodes with low prestige high trust scores. Table 2 shows the bias scores of the nodes for the example in Figure 1 by 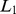-AVG. For fair comparison with MB, we set 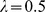 in all of our algorithms in this experiment. We can clearly see that node 5 achieves the highest bias score, which conforms with our intuition. Also, we can observe that 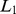-AVG converges in 5 iterations, because the rate of convergence of our framework is exponential.

**Table 2:** Bias scores by the 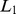-AVG algorithm.

Iteration	node 1	node 2	node 3	node 4	node 5
1	0.115	0.200	0.292	0.111	0.207
2	0.005	0.130	0.137	0.060	0.220
3	0.019	0.117	0.098	0.054	0.233
4	0.018	0.113	0.089	0.054	0.237
5	0.018	0.113	0.089	0.054	0.237

The table shows the bias scores by the 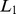-AVG algorithm in the trust network given in Figure 1. The 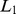-AVG algorithm converges in 5 iterations, and node 5 achieves the highest bias score.

The second 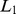-distance based vector-valued contractive function is defined by
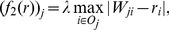
(10)for all 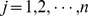. Below, we show that 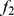 is a vector-valued contractive function.

#### Theorem 3

*For any* 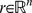, *and* 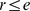, 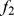 *is a vector-valued contractive function with* 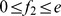.

In 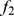, since the bias of node 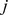 is determined by the maximal difference between the trust scores given by node 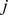 and the corresponding prestige score of the outgoing neighbors of node *j*, we refer to this algorithm as the 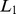 maximal trustworthiness-weighted algorithm (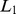-MAX). The corresponding iterative system is as follows.
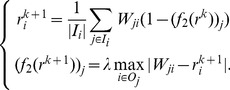
(11)With Eq. (10), we can see that 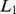-MAX punishes the biased nodes more heavily than 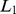-AVG, as it takes the maximal difference to measure the bias. In other words, in 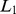-MAX, the node that only gives one unreasonable trust score will get high bias score. Like 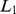-AVG, 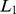-MAX can also prevent attacks from the adversarial nodes who give the nodes with high prestige low trust scores, and give the nodes with low prestige high scores. Table 3 shows the bias scores of the nodes for the example in Figure 1 by 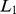-MAX. We can see that node 5 gets the highest bias score as desired. 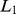-MAX converges in 5 iterations, because the rate of convergence of our framework is exponential.

**Table 3:** Bias scores by the 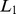-MAX algorithm.

Iteration	node 1	node 2	node 3	node 4	node 5
1	0.115	0.343	0.343	0.165	0.407
2	0.000	0.215	0.215	0.050	0.311
3	0.020	0.179	0.179	0.061	0.289
4	0.017	0.169	0.169	0.065	0.285
5	0.017	0.169	0.169	0.065	0.285

The table shows the bias scores by the 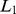-MAX algorithm in the trust network given in Figure 1. The 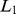-MAX algorithm converges in 5 iterations, and node 5 achieves the highest bias score.

#### 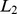 distance based contractive functions

We propose two contractive functions based on the square of 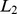 distance measure. For convenience, we refer to these functions as 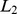 distance based contractive functions. Since the 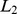 distance based algorithms are defined in a similar fashion as the 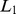 distance based algorithms, we omit explanation unless necessary. The first 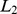 distance based contractive function is given by the following equation.
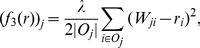
(12)for all 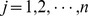. We can also prove the 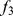 is a vector-valued contractive function.

#### Theorem 4

*For any* 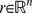, *and* 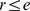, 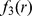 *is a vector-valued contractive function with* 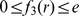.

Similarly, in 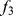, the bias of node 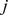 is determined by the arithmetic average of the difference between the trust scores given by node 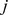 and the corresponding prestige score of the outgoing neighbors of node 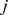. However, unlike 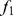 and 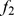, in 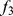, the difference is measured by the square of 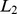 distance. Thus, we refer to this algorithm as the 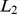 average trustworthiness-weighted algorithm (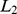-AVG). The corresponding iterative system is
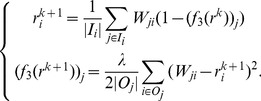
(13)


The second 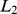 distance based vector-valued contractive function is defined by
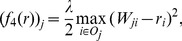
(14)for all 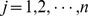. Likewise, we have the following theorem.

#### Theorem 5

*For any* 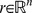, *and* 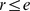, 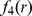 *is a vector-valued contractive function with* 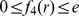.

The corresponding iterative system is
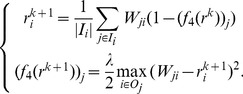
(15)


Similar to 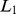-MAX, we refer to this algorithm as the 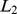 maximal trustworthiness-weighted algorithm (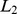-MAX).

We depict the prestige scores by different algorithms in Table 4. We can observe that the rank of the prestige scores by our algorithms is the same as the rank by AA (Arithmetic average) algorithm in Figure 1, and also it is strongly correlated to MB. Note that all of our algorithms give zero prestige score to node 2, as node 2 obtains zero trust score from his/her incoming neighbors. It is worth mentioning that the time and space complexity of all the proposed algorithms are linear, implying that all the algorithms are able to scale to large datasets. The detailed complexity analysis are given in File S1.

**Table 4:** Prestige scores by different algorithms.

Algorithm	node 1	node 2	node 3	node 4	node 5
AA	0.567	0.000	0.433	0.600	0.350
HITS	1.000	0.000	0.401	0.391	0.027
PageRank	0.224	0.030	0.305	0.141	0.300
MB	0.532	0.000	0.433	0.523	0.350
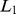 -AVG	0.502	0.000	0.352	0.541	0.336
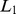 -MAX	0.461	0.000	0.331	0.492	0.335
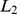 -AVG	0.558	0.000	0.416	0.594	0.349
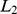 -MAX	0.556	0.000	0.414	0.591	0.348

The table shows the prestige scores by different algorithms in the trust network given in Figure 1.

### Generalizing to signed trust networks

Our algorithms can be generalized to signed trust networks. In signed trust networks, there exist two types of edges: the positive edge and the negative edge. In other words, the weights of positive (negative) edges are positive (negative). In practice, many trust networks, such as Slashdot and Epinions, are signed trust networks, where the negative edges signify distrust. Without loss of generality, we assume that the weights of the edges have been scaled into [−1, 1]. Based on the convergence analysis given in File S1, one can easily show that all the proposed algorithms converge into a unique fixed point in the context of signed trust networks. Moreover, the rate of convergence is exponential. Notice that this result holds if the function 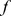 is a vector-valued contractive function. In signed trust networks, it is easy to check that the functions 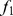 and 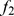 are still the vector-valued contractive functions, but the 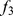 and 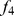 are not. However, we can readily modify them to the vector-valued contractive functions, which are denoted by 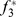 and 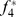 respectively, by adjusting the decay constant. Specifically, we have
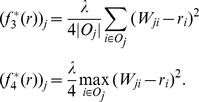
It is easy to verify that 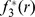 and 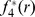 are vector-valued contractive functions in signed trust networks.

## Results

We first briefly describe our experimental settings and then report our findings.

### Setup

We conduct our experiments on five real datasets. (1) Kaitiaki dataset: We collect the Kaitiaki dataset from Trustlet (www.trustlet.org). This dataset is a trust network dataset, where the trust statements are weighted at four different levels (0.4, 0.6, 0.8, and 1.0). (2) Epinions dataset: We download it from Stanford network analysis data collections (http://snap.stanford.edu). It is a signed trust network dataset, where the users can trust or distrust the other users. (3) Slashdot datasets: we collect three different datasets from Stanford network analysis data collections. All of these three datasets are signed trust networks, where the users can give trust or distrust scores to the others. Table 5 summarizes the detailed statistical information of the datasets. We set the decay constant 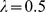 for a fair comparison with MB. For the decay constant of the PageRank algorithm, we set it to 0.85, as it is widely used in web search. All the experiments are conducted on a Windows Server 2008 with 4×6-core Intel Xeon 2.66 Ghz CPU, and 8G memory. All algorithms are implemented by MATLAB 2010a and Visual C++ 6.0.

**Table 5:** Summary of the datasets.

Name	Nodes	Edges	Ref.
Kaitiaki	64	178	website
Epinions	131,828	841,372	[21]
Slashdot1	77,350	516,575	[21]
Slashdot2	81,867	545,671	[21]
Slashdot3	82,140	549,202	[21]

Kaitiaki is a unsigned trust network dataset, while Epinions, Slashdot1, Slashdot2, and Slashdot3 are signed trust network datasets.

### Comparison of bias score

Here we compare the bias scores by our algorithms with the bias scores by MB. First, we use the variance of the trust scores given by node 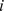 to measure the bias of the node 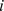, as used in [1]. Specifically, we define the variance as follows:
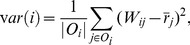
(16)where 
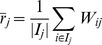
. Second, we rank the nodes by their variance and use this rank as the “ground truth”. Note that there is no ground truth for the bias score of the nodes in any datasets. We use the variance as the ground truth. The reason is twofold. On one hand, the variance is an intuitive metric for measuring the bias of the node, and the node having a larger variance implies that the node has a larger bias score. On the other hand, the variance has been used for analyzing the bias of the node in trust networks [1]. Third, we rank the nodes by their bias scores obtained by our algorithms and obtained by MB, respectively. Specifically, for MB, we rank the nodes by the absolute value of the bias scores (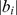 in Eq. (1)). Finally, we compare our algorithms with MB in terms of AUC (the area under the ROC curve) [29] and Kendall Tau [30] metric, where the AUC metric is used to evaluate the top-K rank (in our experiments, we consider the top-5% nodes) and the Kendall Tau metric is employed to evaluate the rank correlation between the rank by the proposed algorithms and the ground truth. Additionally, we remark that, to measure the bias, the variance is based on the average trust score (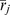), while our algorithms are based on the iteratively refined prestige score. The iteratively refined prestige score is better than the average trust score to reflect the *actual* rank of a node, because the iteratively refined prestige score takes into account the multi-hop neighbors' trust scores. Therefore, in this sense, our proposals are better than the variance to measure the bias of the nodes in trust social networks.

Table 6 and Table 7 show the comparison of bias by our algorithms and MB under AUC and Kendall Tau metric, respectively. From Table 6, we can see that 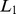-AVG and 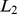-AVG achieve the best performance. In signed trust networks, the performance of our algorithms are significantly better than MB. For example, 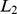-AVG boosts AUC over MB by 4.7%, 11%, 9.9%, and 9.7% in Epinions, Slashdot1, Slashdot2 and Slashdot3, respectively. The results indicate that our algorithms are more effective than MB for computing the bias of the nodes. This is because the bias measurements of our algorithms are more reasonable than the bias measurement of MB. Interestingly, 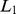-AVG and 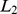-AVG achieve the same performance under the AUC metric. In general, 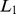-AVG and 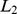-AVG outperform 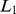-MAX and 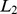-MAX in our datasets. From Table 7, we can observe that all the algorithms exhibit positive correlation to the ground truth. 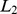-AVG achieves the best performance in Kaitiaki, Epinions, Slashdot1, and Slashdot3 datasets, while in Slashdot2 dataset 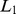-AVG achieves the best performance. It is important to note that all of our algorithms significantly outperform MB in signed networks. For instance, 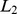-AVG improves Kendall Tau over MB by 11.9%, 6.8%, 10.1%, 12.3%, and 13.9% in Kaitiaki, Epinions, Slashdot1, Slashdot2 and Slashdot3, respectively. The results further confirm that our algorithms are more effective than MB for computing the bias of the node in trust networks.

**Table 6:** Comparison of bias by our algorithms and MB algorithm under AUC metric (top 5% nodes of the dataset).

Datasets	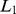 -AVG	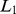 -MAX	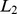 -AVG	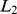 -MAX	MB
Kaitiaki	**1.000**	0.937	**1.000**	0.925	**1.000**
Epinions	**0.994**	0.982	**0.994**	0.982	0.949
Slashdot1	**0.993**	0.970	**0.993**	0.970	0.895
Slashdot2	**0.992**	0.975	**0.992**	0.975	0.903
Slashdot3	**0.992**	0.975	**0.992**	0.975	0.903

The AUC metric is used to measure the top 5% rank of bias by our algorithms and MB algorithm. The larger AUC value implies the better performance.

**Table 7:** Comparison of bias by our algorithms and MB algorithm under Kendall Tau metric.

Datasets	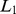 -AVG	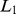 -MAX	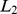 -AVG	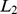 -MAX	MB
Kaitiaki	0.728	0.713	**0.812**	0.709	0.726
Epinions	0.781	0.754	**0.783**	0.754	0.733
Slashdot1	0.811	0.776	**0.812**	0.776	0.734
Slashdot2	**0.722**	0.688	0.721	0.688	0.642
Slashdot3	0.820	0.787	**0.821**	0.787	0.721

The Kendall Tau metric is used to measure the rank of bias by our algorithms and MB algorithm. The larger Kendall Tau value indicates the better performance.

### Comparison of prestige score

This experiment is designed to compare the prestige scores by our algorithms with those by MB. Similarly, there is no ground truth in the datasets, thus we use the rank by the arithmetic average (AA), HITS [18], and PageRank [17] algorithms as the baselines. The reasons are as follows. First, AA, HITS and PageRank algorithms are three widely used ranking algorithms which have been successfully used for measuring the prestige (or centrality) of users in social networks. Second, in singed trust networks, many previous studies [1], [23] have shown that rankings by the HITS and PageRank algorithms and by their signed variants exhibit a very high correlation. For example, in [23], the authors reported that the ranking by the signed spectral ranking algorithm highly correlates with the ranking by the PageRank algorithm. In [1], the authors shown that the ranking by the MB algorithm also highly correlates with the rankings by both HITS and PageRank algorithms. Therefore, in this sense, the HITS and PageRank algorithms can still act as good references for measuring the prestige in signed trust networks.

**Figure 2:**
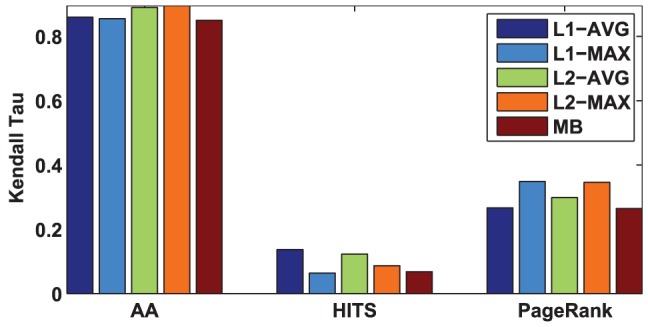
Comparison of prestige by our algorithms and MB algorithm in Kaitiaki dataset. Three methods (AA, HITS, PageRank) are used as baselines for measuring the rank of prestige. The higher Kendall Tau value exhibits higher rank correlation between different algorithms and the baselines.

**Figure 3:**
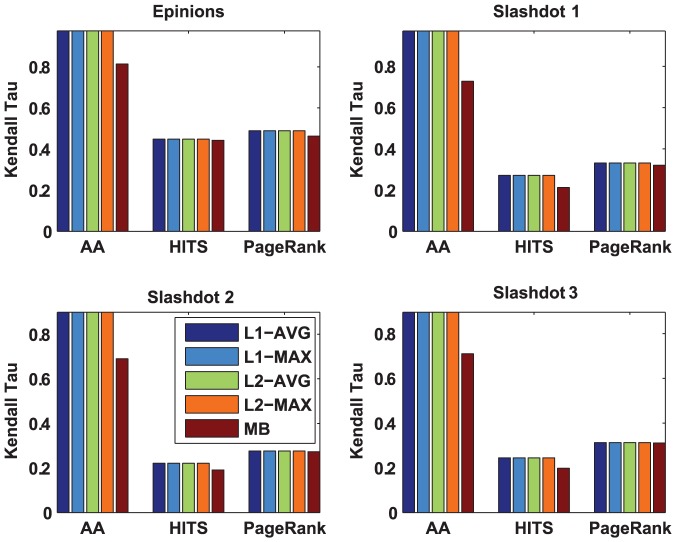
Comparison of prestige by our algorithms and MB algorithm in signed trust networks.

Specifically, we compare the rank correlation between the rank by our algorithms (here we rank the nodes according to their prestige scores) and the rank by the baselines using Kendall Tau metric. Here, AA ranks the nodes by the average trust scores obtained from the incoming neighbors, and HITS ranks the nodes by their authority scores. In signed trust networks, we remove the signed edges for HITS and PageRank, as these algorithms cannot work on signed trust networks directly. Similar evaluation method has been used in [1]. Figure 2 and Figure 3 depict the comparison of prestige score by our algorithms and MB on Kaitiaki and signed trust networks, respectively.

From Figure 2, we can clearly see that our algorithms achieve the best rank correlation to AA. By comparing the Kendall Tau between different algorithms (our algorithms and MB) and HITS, we find that 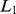-AVG achieves the best rank correlation. However, by comparing the Kendall Tau between different algorithms and PageRank, we clearly find that 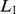-MAX achieves the best rank correlation. From Figure 3, we can also observe that our algorithms achieve the best rank correlation to AA. By comparing the rank correlation between different algorithms and HITS/PageRank, we find that our algorithms are slightly better than MB on the signed trust network datasets. These results suggest that our algorithms are more effective to measure the prestige of the nodes than MB. Interestingly, all of our algorithms achieve the same performance in signed trust networks.

### Robustness testing

To evaluate the robustness of different algorithms, we consider two different types of attacks which could be existent in trust social networks. The first attack model is the *dishonest voting* attack where the dishonest user randomly give high trust score to his/her out-neighbors whose average trust score is low, and randomly give low trust score to his/her outgoing neighbors whose average trust score is high. The second attack model is the *clique* attack where a small group of users form a clique and give the highest trust score to one another so as to increase their prestige scores and decrease their bias scores. The detailed evaluation method is as follows. First, we add some noisy data into the original datasets. Specifically, we randomly select some nodes as the spamming nodes, and then modify the trust scores given by the spamming nodes. In the *dishonest voting* attack model, we revise the trust score given by the spamming nodes as follows. For each spamming node, we randomly give high trust score to his/her out-neighbors whose average trust score is low, and randomly give low trust score to his/her outgoing neighbors whose average trust score is high. In the *clique* attack model, we randomly and evenly partition the spamming nodes into three different types of groups where the size of the first, the second, and the third type of group are 3, 5, and 7 respectively. For instance, if we have selected 30 spamming nodes, then we randomly divide these nodes into 6 groups (i.e., two groups with size 3, two groups with size 5, two groups with size 7). Then, in each group, the nodes give the highest trust score to one another. For two nodes in the same group, if there already exists a trust score, then we revise the trust score by the highest trust score. We have also conducted experiments on other types of group (eg. group with size 10), but the results (not shown) exhibit no significant difference. Second, we perform our algorithms and MB on both original and noisy datasets, and then calculate the Kendall Tau for each algorithm. Here the Kendall Tau is computed on two ranks that are yielded by an algorithm on the original datasets and the noisy datasets, respectively. Finally, we compare the Kendall Tau among all algorithms. Intuitively, the larger Kendall Tau the algorithm achieves, the more robust the algorithm is.

**Figure 4:**
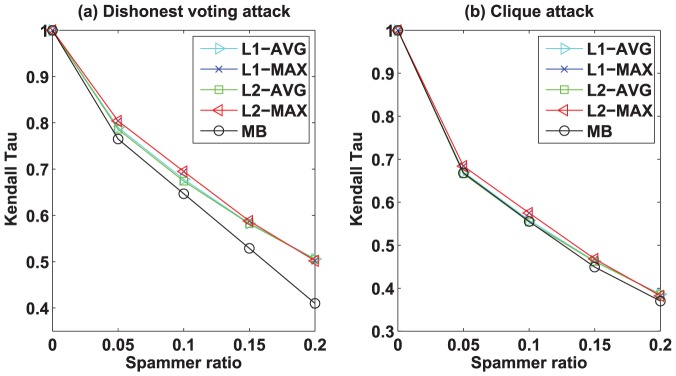
Robustness of bias by our algorithms and MB algorithm in Epinions dataset under (a) *dishonest voting* attack and (b) *clique* attack models. The curves show the robustness of bias by our algorithms and MB algorithm at different spammer ratio. The larger Kendall Tau value implies that the algorithm is more robust. The robustness decreases as the spammer ratio increases. Note that the robustness of our algorithms are consistently better than the MB algorithm under both (a) *dishonest voting* attack and (b) *clique* attack models.

**Figure 5:**
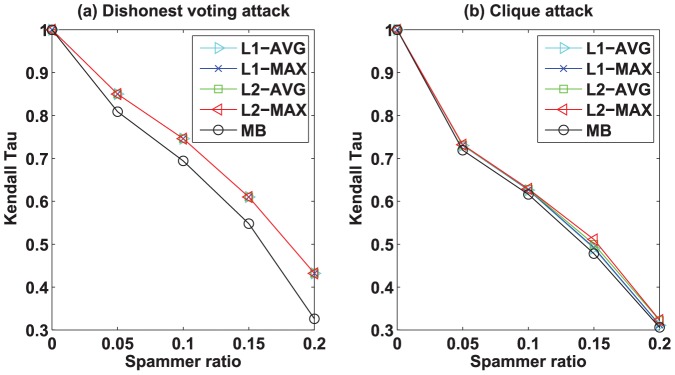
Robustness of prestige by our algorithms and MB algorithm in Epinions dataset under (a) *dishonest voting* attack and (b) *clique* attack models. The curves show the robustness of prestige by our algorithms and MB algorithm at different spammer ratio.

We test our algorithms and MB on both original and noisy datasets with 5% to 20% spamming ratio. Figure 4 and Figure 5 show the robustness of the bias and the prestige of the algorithms by Kendall Tau vs. spamming ratio on Epinions dataset, respectively. Similar results can be obtained from other datasets. First, let us analyze the results by different algorithms under the *dishonest voting* attack. From Figure 4(a) and Figure 5(a), we can clearly see that all of our algorithms are significantly more robust than MB under the *dishonest voting* attack. For the bias, 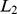-MAX achieves the best robustness, followed by the 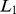-MAX, 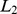-AVG, 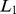-AVG, and then MB. For the prestige, all of our algorithms achieve the same robustness, and are significantly more robust than MB. These results confirm our analysis in the previous section. Moreover, the gap of robustness between our algorithms and MB increases as the spamming ratio increases, which suggests that our algorithms are more effective than MB on the datasets with high spamming ratio. In general, the robustness of the algorithms decrease as the spamming ratio increases. Second, we discuss the results by different algorithms under the *clique* attack. As can be seen from Figure 4(b) and Figure 5(b), our algorithms are slightly better than MB. However, unlike the previous results, the robustness of MB is very close to those of our algorithms. Moreover, we can see that the robustness of all the algorithms under the *clique* attack are worse than the robustness of all the algorithms under the *dishonest voting* attack. For example, in Figure 4, if the spammer ratio is 0.05, the robustness of MB is around 0.75 under the *dishonest voting* attack, while under the *clique* attack the robustness of MB nearly decreases to 0.65. Similar results can be observed for the proposed algorithms. These results indicate that our algorithms and MB could suffer from the *clique* attack. Therefore, designing new algorithms that can defend *clique* attack will be an interesting future direction.

### Scalability

**Figure 6:**
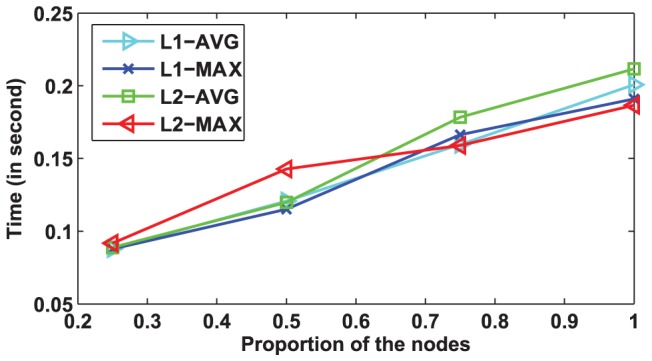
Scalability of the proposed algorithms. The curves show that the running time of our algorithms increases linearly as the number of nodes increases.

We evaluate the scalability of our algorithms on the Epinions dataset. Similar results can be obtained from other datasets. For evaluating the scalability, we first generate three subgraphs in terms of the following rule. First, we randomly select 25% nodes and the corresponding edges of the original graph as the first dataset, and then add another 25% nodes to generate the second dataset, and then based on the second dataset, we add another 25% nodes to generate the third dataset. Then, we perform our algorithms on this three datasets and the original dataset. Figure 6 shows our results. From Figure 6, we can clearly see that our algorithms scales linearly w.r.t. the size of the graph. This result conforms with our complexity analysis in the previous section.

### Effect of parameter 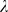


**Figure 7:**
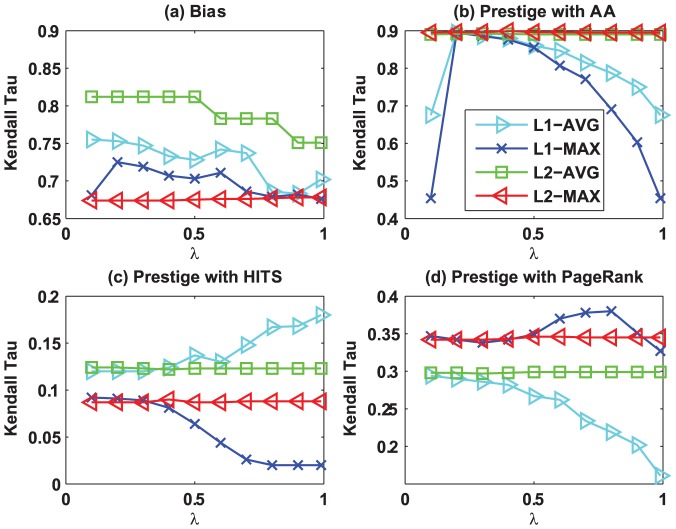
Effect of 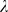. (a) The curves show the bias by our algorithms at different 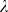. (b–d) The figures show the prestige (compared with different baselines) by our algorithms at different 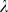 parameter values.

We discuss the effectiveness of parameter 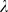 in our algorithms on Kaitiaki dataset. Similar results can be observed from other datasets. Figure 7 shows the effectiveness of our algorithms w.r.t. 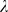, where the effectiveness is measured by the rank correlation between our algorithms and the baselines using the Kendall Tau metric. Specifically, Figure 7(a) depicts the bias correlation between our algorithms and the *variance* based algorithm (Eq. (16)) under various 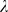, while Figure 7(b), (c), and (d) show the prestige correlation between our algorithms and AA, HITS, and PageRank under different 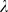, respectively. From Figure 7(a), we find that 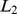-MAX is quite robust w.r.t. 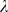, while the performance of 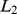-AVG decreases as 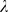 increases. In addition, we find that 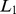-AVG and 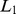-MAX are slightly sensitive w.r.t. 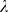, because the differences between the maximal and minimal bias correlation of these two algorithms do not exceed 0.1. For the prestige scores (Figure 7(b), (c), and (d)), we can clearly see that 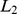-AVG and 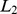-MAX are more robust w.r.t. 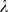, whereas 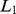-AVG and 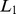-MAX are sensitive w.r.t. 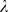. For instance, consider the prestige correlation with PageRank (Figure 7(d)), we can observe that the performance of 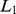-AVG decreases as 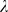 increases. However, the performance of 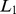-MAX increases as 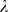 increases when 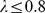, and otherwise it decreases as 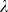 increases. To summarize, the 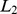 distance based algorithms are more robust w.r.t. the parameter 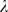 than the 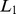 distance based algorithms.

## Discussion

Bias and prestige are two essential features in trust networks, therefore it is crucial to have an efficient and effective algorithm to compute them. In this paper, we define a vector-valued contractive function to characterize the bias vector for every node in the trust network. Based on this, we propose a framework of algorithms for computing the bias and prestige of nodes in trust networks in an iterative way. The proposed framework allows us to develop new bias measures which can circumvent the major drawbacks in the existing algorithm. Moreover, our framework can converges into a unique and fixed point with an exponential rate. We believe that the proposed framework can be used to measure and analyze the bias and prestige of nodes in trust networks, which could be very useful for trust-based recommendation systems and many other trust-based application domains.

There are several open questions that are deserved to further investigation. First, all of our algorithms currently only work on static trust networks. However, many real-world trust social networks evolve over time, thereby it remains a challenging problem to generalize our framework to time-evolving trust networks. Recently, some proposals on incremental PageRank algorithm have been proposed [31], [32]. Similar ideas could be also used to devise incremental counterparts of our algorithms. Second, trust social network is a decentralized social system, where the users can only interact with their immediate neighbors. In such decentralized social systems, an interesting question is that whether or not a user in trust social networks can estimate his/her global prestige and bias scores by only using the local trust scores. To answer this question, one potential solution is to extend our framework to a decentralized one. The ideas from gossip-based algorithms such as [33], [34] could be used to solve this problem. Finally, as shown in the experiments, the proposed algorithms and MB suffer from the *clique* attack. Therefore, devising robust algorithms that can defend such *clique* attacks would be an interesting future direction.

## Supporting Information

File S1 **Supplementary document.**
(PDF)
