# Correction: A Framework of Algorithms: Computing the Bias and Prestige of Nodes in Trust Networks

**DOI:** 10.1371/annotation/e57b110b-249b-4963-8b2c-53fc48c43de4

**Published:** 2013-05-21

**Authors:** Rong-Hua Li, Jeffrey Xu Yu, Xin Huang, Hong Cheng

There was an error in the Funding Statement. Grant number 419109 is incorrect. The correct grant number is 418512. 

