# Supplementary material for: A Framework of Algorithms: Computing the Bias and Prestige of Nodes in Trust Networks
Source: PLoS One. 2012 Dec 11;7(12):e50843. doi: 10.1371/journal.pone.0050843 (PMC3519843; doi:10.1371/journal.pone.0050843)
Supplement: File S1 — Supplementary document. (PDF) [file pone.0050843.s001.pdf]

Supplementary document

Rong-Hua Li, Jeffrey Xu Yu, Xin Huang, Hong Cheng
Department of Systems Engineering & Engineering Management, The Chinese University of Hong Kong, Sha Tin, N.T., Hong Kong.
∗ E-mail: rhli@se.cuhk.hk.edu

1 Analysis of the proposed framework

Convergence of the proposed framework: We analyze the convergence properties of the following iterative system

$$\begin{cases} r_i^{k+1} = \frac{1}{|I_i|}\sum\limits_{j\in I_i} W_{ji}(1-b_j^k) \\ b_j^{k+1} = (f(r^{k+1}))_j \end{cases} \tag{1}$$

Specifically, we show the prestige vector will converge into a unique fixed point as stated in Theorem 1. Similar arguments can be used to prove the bias vector also converges into a unique fixed point. First, we prove the following lemma.

Lemma 1: *For any node i, $|r_i^{k+1} - r_i^k| \le \lambda^k ||r^1 - r^0||_\infty$.*
Proof: We prove Lemma 1 by induction. Let $k = 1$, we have

$$\begin{aligned} |r_i^2 - r_i^1| &= |\tfrac{1}{|I_i|}\sum_{j\in I_i} W_{ji}((f(r^0))_j - (f(r^1))_j)| \\ &\le \tfrac{1}{|I_i|}\sum_{j\in I_i} W_{ji}|(f(r^0))_j - (f(r^1))_j| \\ &\le \tfrac{\lambda}{|I_i|}\sum_{j\in I_i} W_{ji}||r^1 - r^0||_\infty \\ &\le \lambda||r^1 - r^0||_\infty, \end{aligned}$$

where the second inequality is due to the definition of vector-valued contractive function, and the last inequality is by $|W_{ij}| \in [0, 1]$. Assume the lemma holds when $k = t$. We show that the lemma still holds when $k = t + 1$.

$$\begin{aligned} |r_i^{t+2} - r_i^{t+1}| &= |\tfrac{1}{|I_i|}\sum_{j\in I_i} W_{ji}((f(r^t))_j - (f(r^{t+1}))_j)| \\ &\le \tfrac{1}{|I_i|}\sum_{j\in I_i} W_{ji}|(f(r^t))_j - (f(r^{t+1}))_j| \\ &\le \tfrac{\lambda}{|I_i|}\sum_{j\in I_i} W_{ji}||r^{t+1} - r^t||_\infty \\ &\le \lambda||r^{t+1} - r^t||_\infty \\ &\le \lambda^{t+1}||r^1 - r^0||_\infty, \end{aligned}$$

where the second inequality is due to the definition of vector-valued contractive function and the last inequality holds by the induction assumption. This completes the proof. □

With Lemma 1, we prove the convergence property.

Theorem 1: *The iterative system defined in Eq. (1) converges into a unique fixed point.*
Proof: We first prove the convergence of the iterative system (Eq. (1)), and then prove the uniqueness. Specifically, for $\varepsilon > 0$, there exists N such that

$$\lambda^N < \frac{(1-\lambda)\varepsilon}{||r^1 - r^0||_\infty}.$$

Then, for any $s > t \ge N$, we have

$$
\begin{aligned}
|r_i^s - r_i^t| &\le |r_i^s - r_i^{s-1}| + |r_i^{s-1} - r_i^{s-2}| + \cdots + |r_i^{t+1} - r_i^t| \\
&\le \lambda^{s-1}||r^1 - r^0||_\infty + \lambda^{s-2}||r^1 - r^0||_\infty + \cdots + \\
&\quad \lambda^t||r^1 - r^0||_\infty \\
&\le ||r^1 - r^0||_\infty \lambda^t \sum_{k=0}^{s-t-1} \lambda^k < ||r^1 - r^0||_\infty \lambda^t \sum_{k=0}^{\infty} \lambda^k \\
&= ||r^1 - r^0||_\infty \lambda^t \tfrac{1}{1-\lambda} \\
&\le ||r^1 - r^0||_\infty \lambda^N \tfrac{1}{1-\lambda} \\
&\le \varepsilon,
\end{aligned}
$$

where the first inequality holds by the triangle inequality, and the second inequality is due to Lemma 1. Then, by Cauchy convergence theorem [1], we conclude that the sequence r_α^k converges to a fixed point. For the uniqueness, we prove it by contradiction. Suppose Eq. (1) has at least two fixed points. Let $r^{(1)}$ and $r^{(2)}$ be two fixed points, and $M = |r_i^{(1)} - r_i^{(2)}| = ||r^{(1)} - r^{(2)}||_\infty$. Then, we have

$$
\begin{aligned}
M &= |\tfrac{1}{|I_i|} \sum_{j \in I_i} W_{ji}((f(r^{(1)}))_j - (f(r^{(2)}))_j)| \\
&\le \tfrac{1}{|I_i|} \sum_{j \in I_i} W_{ji}|((f(r^{(1)}))_j - (f(r^{(2)}))_j)| \\
&\le \tfrac{\lambda}{|I_i|} \sum_{j \in I_i} W_{ji}||r^{(1)} - r^{(2)}||_\infty \\
&\le \lambda||r^{(1)} - r^{(2)}||_\infty = \lambda M.
\end{aligned}
$$

Since $\lambda \in [0, 1)$, thus $M < M$, which is a contradiction. This completes the proof. □

The rate of convergence: We show that our framework will converge in exponential rate by the following lemmas.

Lemma 2: $||r^\infty - r^k||_\infty \le \lambda^k||r^\infty - r^0||_\infty$.
Proof: We prove the lemma by induction. For $k = 1$, let $|r_i^\infty - r_i^1| = ||r^\infty - r^1||_\infty$, then we have

$$
\begin{aligned}
|r_i^\infty - r_i^1| &= |\tfrac{1}{|I_i|} \sum_{j \in I_i} W_{ji}((f(r^0))_j - (f(r^\infty))_j)| \\
&\le \tfrac{1}{|I_i|} \sum_{j \in I_i} W_{ji}|(f(r^0))_j - (f(r^\infty))_j)| \\
&\le \tfrac{\lambda}{|I_i|} \sum_{j \in I_i} W_{ji}||r^\infty - r^0||_\infty \le \lambda||r^\infty - r^0||_\infty
\end{aligned}
$$

The last inequality holds by the definition of vector-valued contractive function. Suppose $k = t$, we have $||r^\infty - r^t||_\infty \le \lambda^t||r^\infty - r^0||_\infty$. Then, when $k = t + 1$, for any node u of the graph, we have

$$
\begin{aligned}
|r_u^\infty - r_u^{t+1}| &= |\tfrac{1}{|I_u|} \sum_{j \in I_u} W_{ju}((f(r^t))_j - (f(r^\infty))_j)| \\
&\le \tfrac{1}{|I_u|} \sum_{j \in I_u} W_{ju}|(f(r^t))_j - (f(r^\infty))_j)| \\
&\le \tfrac{\lambda}{|I_u|} \sum_{j \in I_u} W_{ju}||r^\infty - r^t||_\infty \\
&\le \lambda||r^\infty - r^t||_\infty \le \lambda^{t+1}||r^\infty - r^0||_\infty.
\end{aligned}
$$

Thus, we have $||r^\infty - r^t||_\infty \le \lambda^{t+1}||r^\infty - r^0||_\infty$. This completes the proof. □

Lemma 3: $||r^a - r^b||_\infty \le 1$.
Proof: By definition, for any t, $f(r^t) \le e$ holds. Thus, we conclude $||r^a - r^b||_\infty \le 1$. □

With the above lemma, we readily have the following corollary.

Corollary 1: $||r^\infty - r^k||_\infty \le \lambda^k$.

By Corollary 1, our algorithms converge in exponential rate. We can determine the maximal steps that are needed for convergence. Assume r_i is the true prestige score of node i. Our goal is to show that

after a particular number of iterations k, the prestige score given by our algorithm converges to r_i as desired. Formally, for $\varepsilon \to 0$, let $|r_i - r_i^k| \leq \varepsilon$. By Corollary 1, we can set

$$k = \log_\lambda \varepsilon. \tag{2}$$

This implies that the number of iterations k is a very small constant to guarantee convergence of our framework.

2 Other missing proofs

Theorem 2: *For any $r \in \mathbb{R}^n$, and $r \leq e$, f_{mb} is a vector-valued contractive function with the decay constant $\lambda = 1/2$ and $0 \leq f_{mb} \leq e$.*
Proof: For any $r, s \in \mathbb{R}^n$ and j, let

$$\begin{aligned}\Delta_j &= |(f_{mb}(r))_j - (f_{mb}(s))_j| \\ &= |\max\{0, \tfrac{1}{2|O_j|} \sum_{i \in O_j} (W_{ji} - r_i)\} - \\ &\quad \max\{0, \tfrac{1}{2|O_j|} \sum_{i \in O_j} (W_{ji} - s_i)\}|.\end{aligned}$$

Consider the following four cases:
(A) $\frac{1}{2|O_j|} \sum_{i \in O_j} (W_{ji} - r_i) \leq 0$ and $\frac{1}{2|O_j|} \sum_{i \in O_j} (W_{ji} - s_i) \leq 0$. Obviously, $\Delta_j = 0 \leq \frac{1}{2}||r - s||_\infty$.
(B) $\frac{1}{2|O_j|} \sum_{i \in O_j} (W_{ji} - r_i) \geq 0$ and $\frac{1}{2|O_j|} \sum_{i \in O_j} (W_{ji} - s_i) \geq 0$. We have

$$\begin{aligned}\Delta_j &= |\tfrac{1}{2|O_j|} \sum_{i \in O_j} (s_i - r_i)| \\ &\leq \tfrac{1}{2|O_j|} \sum_{i \in O_j} |s_i - r_i| \\ &\leq \tfrac{1}{2|O_j|} \sum_{i \in O_j} ||r - s||_\infty \\ &= \tfrac{1}{2}||r - s||_\infty.\end{aligned}$$

(C) $\frac{1}{2|O_j|} \sum_{i \in O_j} (W_{ji} - r_i) \geq 0$ and $\frac{1}{2|O_j|} \sum_{i \in O_j} (W_{ji} - s_i) \leq 0$. By $\frac{1}{2|O_j|} \sum_{i \in O_j} (W_{ji} - s_i) \leq 0$, we have $\sum_{i \in O_j} W_{ji} \leq \sum_{i \in O_j} s_i$. Then, we have

$$\begin{aligned}\Delta_j &= \tfrac{1}{2|O_j|} \sum_{i \in O_j} (W_{ji} - r_i) \\ &\leq \tfrac{1}{2|O_j|} \sum_{i \in O_j} (s_i - r_i) \\ &\leq \tfrac{1}{2|O_j|} \sum_{i \in O_j} |s_i - r_i| \\ &\leq \tfrac{1}{2}||r - s||_\infty.\end{aligned}$$

(D) $\frac{1}{2|O_j|} \sum_{i \in O_j} (W_{ji} - r_i) \leq 0$ and $\frac{1}{2|O_j|} \sum_{i \in O_j} (W_{ji} - s_i) \geq 0$. Similar to the case (3), we have $\Delta_j \leq \frac{1}{2}||r - s||_\infty$.
To summarize, for any j, we have $\Delta_j \leq \frac{1}{2}||r - s||_\infty$. Hence, f_{mb} is a vector-valued contractive function with $\lambda = 1/2$. Since $0 \leq W_{ji} \leq 1$ and $r \leq e$, thus $0 \leq f_{mb} \leq e$. This completes the proof. □

Theorem 3: *For any $r \in \mathbb{R}^n$, and $r \leq e$, f_1 is a vector-valued contractive function with $0 \leq f_1 \leq e$.*

Proof: For any $r, s \in \mathbb{R}^n$, we have

$$\begin{aligned}
&|(f_1(r))_j - (f_1(s))_j| \\
&= |\tfrac{\lambda}{|O_j|} \sum_{i \in O_j} |W_{ji} - r_i| - \tfrac{\lambda}{|O_j|} \sum_{i \in O_j} |W_{ji} - s_i|| \\
&= \tfrac{\lambda}{|O_j|} | \sum_{i \in O_j} (|W_{ji} - r_i| - |W_{ji} - s_i|)| \\
&\leq \tfrac{\lambda}{|O_j|} \sum_{i \in O_j} |r_i - s_i| \\
&\leq \lambda ||r - s||_\infty
\end{aligned}$$

Since $0 \leq r \leq e$, $0 \leq W_{ji} \leq 1$ and $0 \leq \lambda < 1$, thus $0 \leq f_1 \leq e$. □

Theorem 4: *For any $r \in \mathbb{R}^n$, and $r \leq e$, f_2 is a vector-valued contractive function with $0 \leq f_2 \leq e$.*

Proof: For any $r, s \in \mathbb{R}^n$, let $|W_{ju} - r_u| = \max_{i \in O_j} |W_{ji} - r_i|$, and $|W_{jv} - s_v| = \max_{i \in O_j} |W_{ji} - s_i|$, then we have

$$\begin{aligned}
&|(f_2(r))_j - (f_2(s))_j| \\
&= |\lambda \max_{i \in O_j} |W_{ji} - r_i| - \lambda \max_{i \in O_j} |W_{ji} - s_i|| \\
&\leq \lambda \max\{||W_{ju} - r_u| - |W_{ju} - s_u||, ||W_{jv} - r_v| - |W_{jv} - s_v||\} \\
&\leq \lambda \max\{|r_u - s_u|, |r_v - s_v|\} \\
&\leq \lambda ||r - s||_\infty
\end{aligned}$$

Since $0 \leq r \leq e$, $0 \leq W_{ji} \leq 1$ and $0 \leq \lambda < 1$, thus $0 \leq f_2 \leq e$. □

Theorem 5: *For any $r \in \mathbb{R}^n$, and $r \leq e$, $f_3(r)$ is a vector-valued contractive function with $0 \leq f_3(r) \leq e$.*

Proof: For any $r, s \in \mathbb{R}$, and $r \leq e, s \leq e$, we have

$$\begin{aligned}
&|(f_3(r))_j - (f_3(s))_j| \\
&= |\tfrac{\lambda}{2|O_j|} \sum_{i \in O_j} (W_{ji} - r_i)^2 - \tfrac{\lambda}{2|O_j|} \sum_{i \in O_j} (W_{ji} - s_i)^2| \\
&\leq \tfrac{\lambda}{2|O_j|} \sum_{i \in O_j} |(W_{ji} - r_i)^2 - (W_{ji} - s_i)^2| \\
&= \tfrac{\lambda}{2|O_j|} \sum_{i \in O_j} |(s_i - r_i)(2W_{ji} - r_i - s_i)| \\
&\leq \tfrac{\lambda}{|O_j|} \sum_{i \in O_j} |s_i - r_i| \\
&\leq \lambda ||r - s||_\infty
\end{aligned}$$

Since $0 \leq r \leq e$, $0 \leq W_{ji} \leq 1$ and $0 \leq \lambda < 1$, thus $0 \leq f_3 \leq e$. □

Theorem 6: *For any $r \in \mathbb{R}^n$, and $r \leq e$, $f_4(r)$ is a vector-valued contractive function with $0 \leq f_4(r) \leq e$.*

Proof: For any $r, s \in \mathbb{R}$, and $r \leq e, s \leq e$, let $(W_{ju} - r_u)^2 = \max_{i \in O_j} (W_{ji} - r_i)^2$, and $(W_{jv} - s_v)^2 = \max_{i \in O_j} (W_{ji} - s_i)^2$, then we have

$$\begin{aligned}
&|(f_4(r))_j - (f_4(s))_j| \\
&= |\tfrac{\lambda}{2} \max_{i \in O_j} (W_{ji} - r_i)^2 - \tfrac{\lambda}{2} \max_{i \in O_j} (W_{ji} - s_i)^2| \\
&\leq \tfrac{\lambda}{2} \max\{|(W_{ju} - r_u)^2 - (W_{ju} - s_u)^2|, \\
&\qquad |(W_{jv} - s_v)^2 - (W_{jv} - r_v)^2|\} \\
&= \tfrac{\lambda}{2} \max\{|(s_u - r_u)(2W_{ju} - r_u - s_u)|, \\
&\qquad |(s_v - r_v)(2W_{jv} - r_v - s_v)|\} \\
&\leq \lambda \max\{|s_u - r_u|, |s_v - r_v|\} \\
&\leq \lambda ||r - s||_\infty
\end{aligned}$$

Since $0 \leq r \leq e$, $0 \leq W_{ji} \leq 1$ and $0 \leq \lambda < 1$, thus $0 \leq f_4 \leq e$. $\square$

3 Complexity of the proposed algorithms

We analyze the time and space complexities of L_1-AVG. For the other algorithms, it is not hard to show that the time and space complexities are the same as L_1-AVG. First, the time complexity for computing the prestige score of node i in one iteration is $O(|\bar{I}||\bar{O}|)$, where $|\bar{I}|$ and $|\bar{O}|$ denote the average in-degree and out-degree of all nodes respectively. The amortized time complexity in one iteration is $O(m)$, where m denotes the number of edges in the graph. Therefore, the total time complexity of L_1-AVG is $O(km)$, where k denotes the number of iterations that are needed to guarantee convergence. As analyzed in Section 1, k is a very small constant. And $k = 15$ can guarantee the algorithms converge as shown in our experiments. The analysis implies that the time complexity of our algorithms is linear w.r.t. the size of the graph. Second, we only need to store the graph, the prestige vector (r), and the contractive function $f(r)$, thus the space complexity is $O(m + n)$. In summary, our algorithms have linear time and space complexities, thereby they can be scalable to large graphs.

References

1. Apostol TM (1974) Mathematical Analysis. Addison Wesley; 2nd edition.
